# Supplementary material for: A Humanized Yeast Phenomic Model of Deoxycytidine Kinase to Predict Genetic Buffering of Nucleoside Analog Cytotoxicity
Source: Genes (Basel). 2019 Sep 30;10(10):770. doi: 10.3390/genes10100770 (PMC6826991; doi:10.3390/genes10100770)
Supplement: Supplementary file 1 [file genes-10-00770-s001.zip › Additional_Files/Additional_File7_GOTermHeatmaps.zip!/Additional_File7_GOTermHeatmaps/cell_division.pdf]

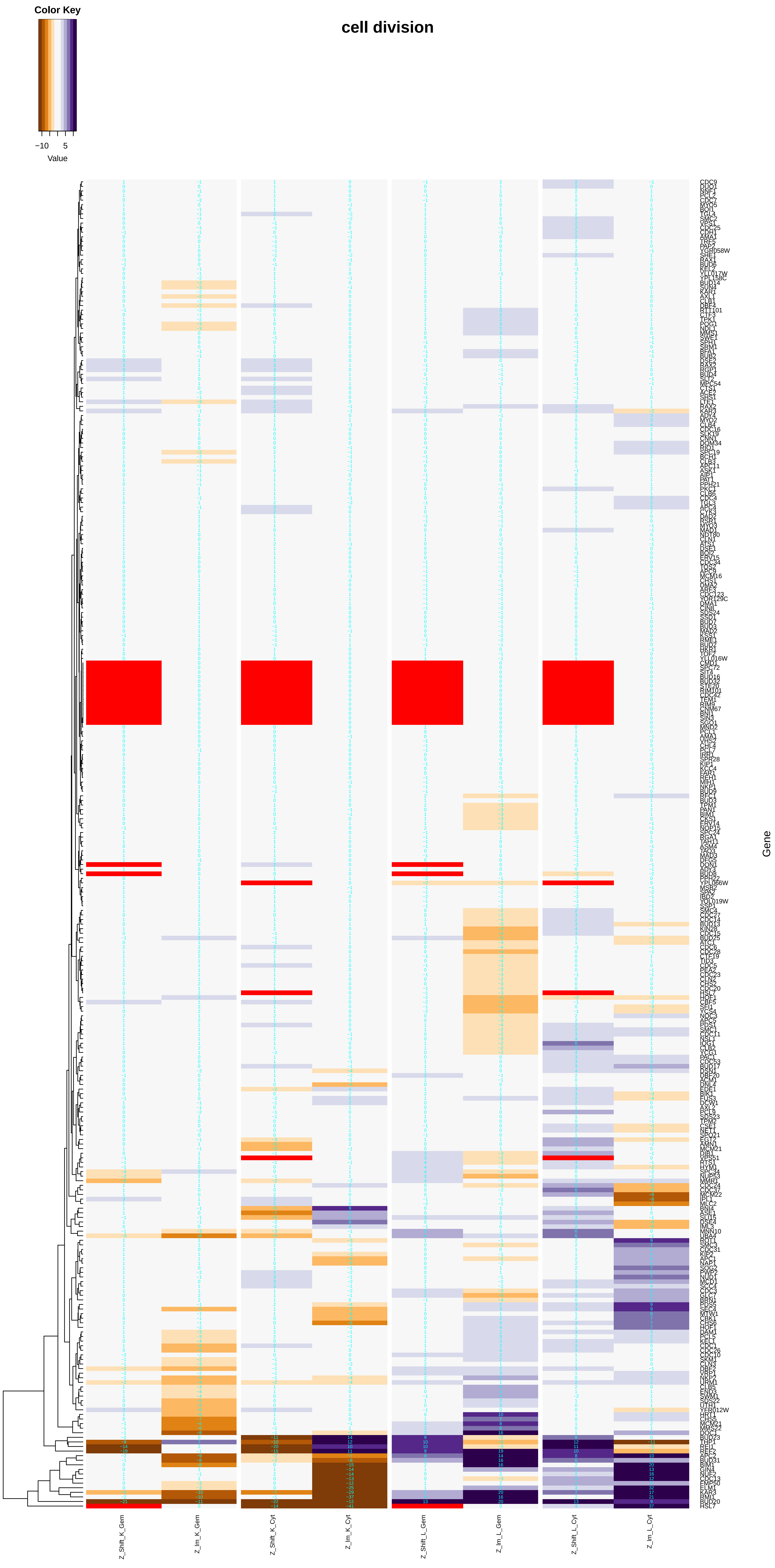
Color Key
−10
5
Value
cell division
Gene
Z_Shift_K_Gem
Z_Im_K_Gem
Z_Shift_K_Cyt
Z_Im_K_Cyt
Z_Shift_L_Gem
Z_Im_L_Gem
Z_Shift_L_Cyt
Z_Im_L_Cyt

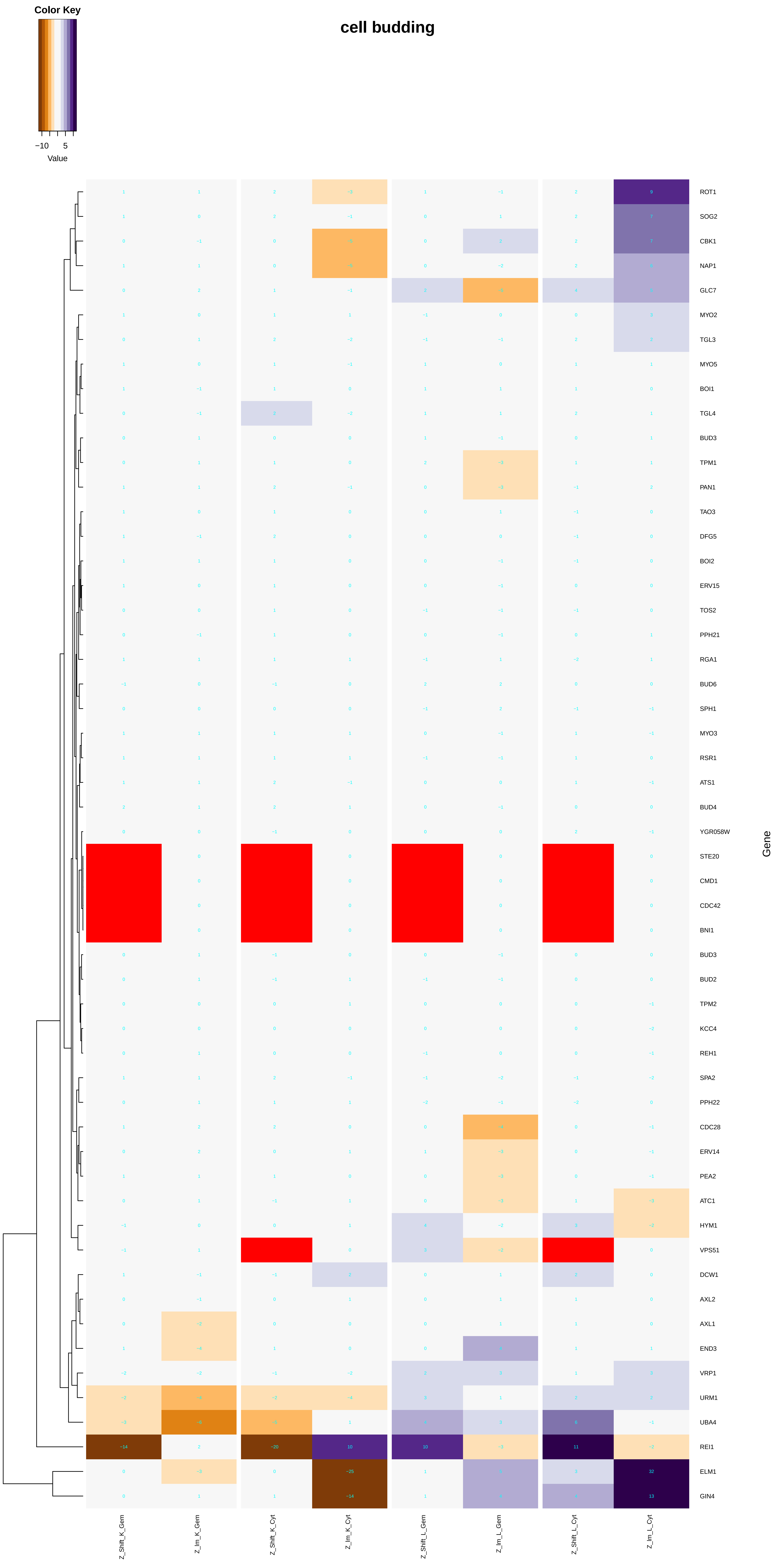
Color Key
−10
5
Value
cell budding
ROT1
SOG2
CBK1
NAP1
GLC7
MYO2
TGL3
MYO5
BOI1
TGL4
BUD3
TPM1
PAN1
TAO3
DFG5
BOI2
ERV15
TOS2
PPH21
RGA1
BUD6
SPH1
MYO3
RSR1
ATS1
BUD4
YGR058W
STE20
CMD1
CDC42
BNI1
BUD3
BUD2
TPM2
KCC4
REH1
SPA2
PPH22
CDC28
ERV14
PEA2
ATC1
HYM1
VPS51
DCW1
AXL2
AXL1
END3
VRP1
URM1
UBA4
REI1
ELM1
GIN4
Gene
Z_Shift_K_Gem
Z_lm_K_Gem
Z_Shift_K_Cyt
Z_lm_K_Cyt
Z_Shift_L_Gem
Z_lm_L_Gem
Z_Shift_L_Cyt
Z_lm_L_Cyt

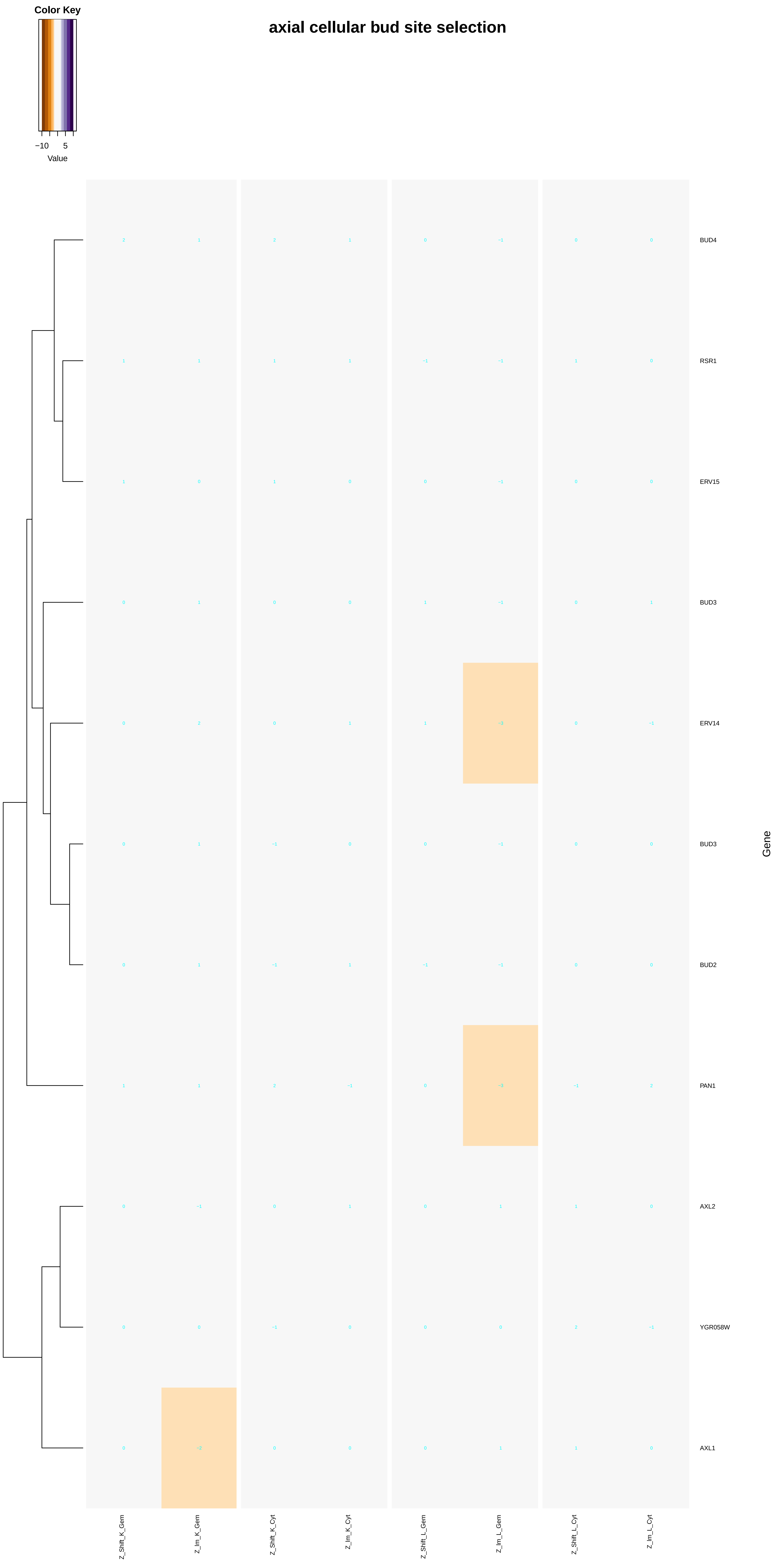
axial cellular bud site selection
Color Key
−10
5
Value
BUD4
RSR1
ERV15
BUD3
ERV14
BUD3
BUD2
PAN1
AXL2
YGR058W
AXL1
Gene
Z_Shift_K_Gem
Z_Im_K_Gem
Z_Shift_K_Cyt
Z_Im_K_Cyt
Z_Shift_L_Gem
Z_Im_L_Gem
Z_Shift_L_Cyt
Z_Im_L_Cyt

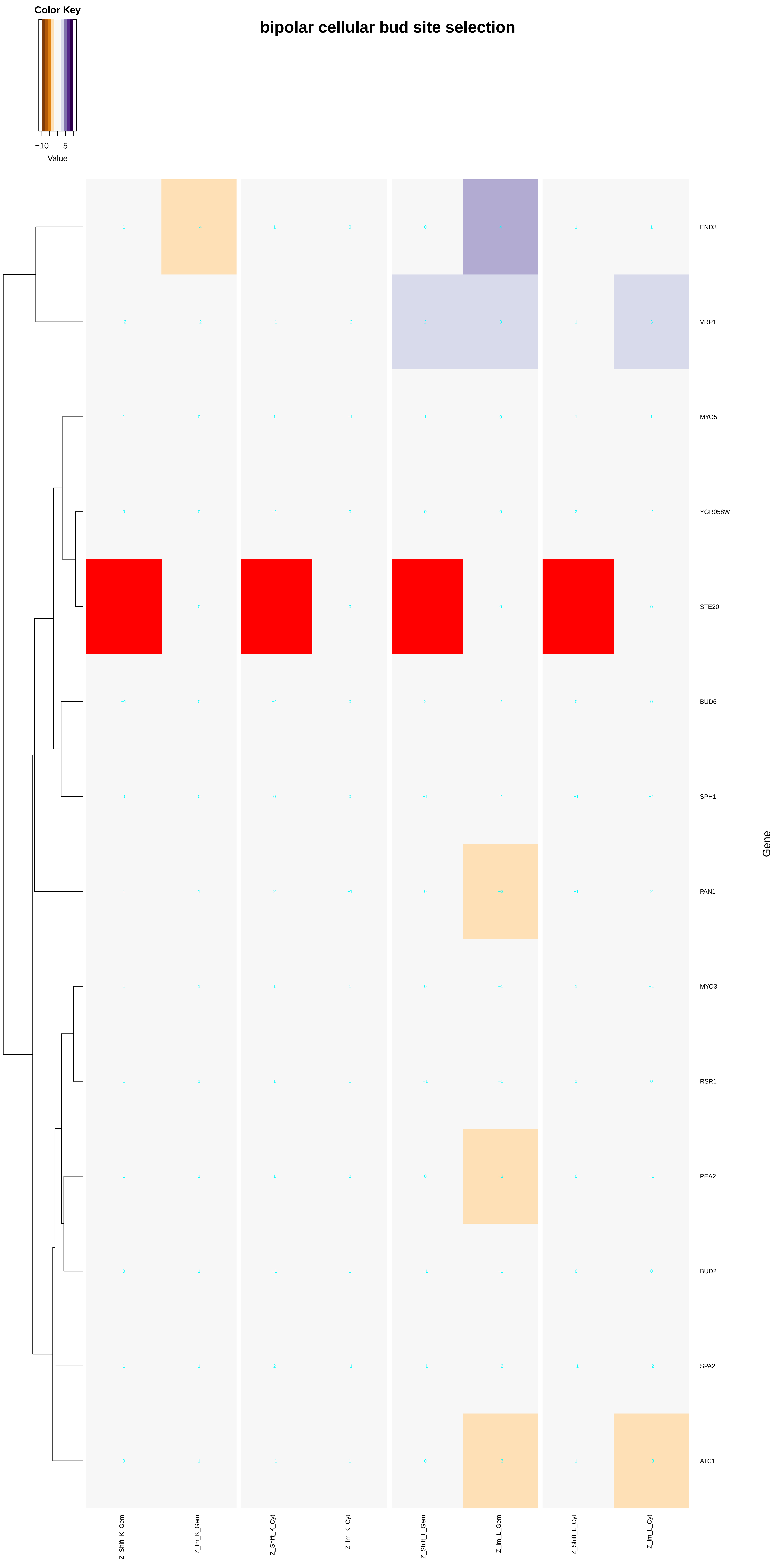
Color Key
−10
5
Value
bipolar cellular bud site selection
END3
VRP1
MYO5
YGR058W
STE20
BUD6
SPH1
PAN1
MYO3
RSR1
PEA2
BUD2
SPA2
ATC1
Gene
Z_Shift_K_Gem
Z_Im_K_Gem
Z_Shift_K_Cyt
Z_Im_K_Cyt
Z_Shift_L_Gem
Z_Im_L_Gem
Z_Shift_L_Cyt
Z_Im_L_Cyt
